# Mitochondrial Regulation of the Muscle Microenvironment in Critical Limb Ischemia

**DOI:** 10.3389/fphys.2015.00336

**Published:** 2015-11-18

**Authors:** Terence E. Ryan, Cameron A. Schmidt, Tom D. Green, David A. Brown, P. Darrell Neufer, Joseph M. McClung

**Affiliations:** ^1^Department of Physiology, Brody School of Medicine, East Carolina UniversityGreenville, NC, USA; ^2^East Carolina Diabetes and Obesity Institute, Brody School of Medicine, East Carolina UniversityGreenville, NC, USA

**Keywords:** skeletal muscle, vascular diseases, mitochondria, ischemia, peripheral arterial disease, angiogenesis

## Abstract

Critical limb ischemia (CLI) is the most severe clinical presentation of peripheral arterial disease and manifests as chronic limb pain at rest and/or tissue necrosis. Current clinical interventions are largely ineffective and therapeutic angiogenesis based trials have shown little efficacy, highlighting the dire need for new ideas and novel therapeutic approaches. Despite a decade of research related to skeletal muscle as a determinant of morbidity and mortality outcomes in CLI, very little progress has been made toward an effective therapy aimed directly at the muscle myopathies of this disease. Within the muscle cell, mitochondria are well positioned to modulate the ischemic cellular response, as they are the principal sites of cellular energy production and the major regulators of cellular redox charge and cell death. In this mini review, we update the crucial importance of skeletal muscle to CLI pathology and examine the evolving influence of muscle and endothelial cell mitochondria in the complex ischemic microenvironment. Finally, we discuss the novelty of muscle mitochondria as a therapeutic target for ischemic pathology in the context of the complex co-morbidities often associated with CLI.

## Introduction

Peripheral artery disease (PAD) presents as either symptom-free, intermittent claudication (IC, pain with exertion that is relieved with rest) or critical limb ischemia (CLI, pain at rest with or without tissue necrosis or gangrene). CLI carries alarmingly high morbidity and mortality rates and patients have a risk of major amputation or death that approaches 40% in 1 year (Dormandy et al., 1999; Hirsch et al., 2001; Taylor et al., 2009). A common misconception is that CLI represents the natural progression of IC in patients; however the same degree of stenosis can present as symptom-free, IC, or CLI, implying that factors other than limb blood flow contribute to pathology. Despite recent advances in stem cell biology and genetics (Matzke and Lepantalo, 2001; Chalothorn et al., 2007; Dokun et al., 2008; Chalothorn and Faber, 2010; Wang et al., 2010, 2012; Katwal and Dokun, 2011), surprisingly little progress has been made toward effective therapeutic options for CLI, warranting the consideration of alternative and novel treatment approaches. Limb skeletal muscle is uniquely positioned to alter the clinical course of CLI due to its inherent plasticity, role as a paracrine signaling organ, and reservoir of endogenous pluripotent progenitor cells (Seale et al., 2001; Chargé and Rudnicki, 2004; Abou-Khalil et al., 2010). Currently, PAD research is overwhelmingly focused on limb collateral vessel development and nascent conduit promotion and survival (Annex, 2013), while potential alternative therapies directed at limb muscle in CLI have been slow to develop. In this mini-review we highlight the importance of skeletal muscle in the manifestation of CLI and discuss the potential influence of muscle and endothelial cell mitochondria on the ischemic limb.

## Skeletal muscle pathology in the ischemic limb

Variations in the clinical course of CLI raise the intriguing possibility that disease manifestation is in part dependent on genetic determinants of susceptibility to ischemia (Matzke and Lepantalo, 2001; Chalothorn et al., 2007; Dokun et al., 2008; Chalothorn and Faber, 2010; Wang et al., 2010, 2012; Katwal and Dokun, 2011). The genetics of PAD are not well understood (Gudmundsson et al., 2002; Knowles et al., 2007; Messina, 2008; Katwal and Dokun, 2011; Leeper et al., 2012; Murabito et al., 2012) but present a complicated paradigm whereby differential determinants could direct the ischemic responses of multiple cell types (endothelial, muscle, fibroblast, etc.) in the affected limb. In this regard, inbred mouse strains have dramatically different responses to a murine model of PAD, analogous to the range of responses seen in humans. For example, limb perfusion recovers rapidly and without tissue loss in C57BL/6J (BL6) mice while BALB/cJ mice display significant tissue necrosis and poor perfusion recovery (Chalothorn et al., 2007; Dokun et al., 2008; Chalothorn and Faber, 2010; Wang et al., 2010). Inherent genetic differences in muscle regeneration are known to occur in BALB/cJ mice (Grounds, 1987; Grounds and McGeachie, 1989; McGeachie and Grounds, 1995; Mitchell et al., 1995; Roberts et al., 1997; Lagrota-Candido et al., 2010), and includes temporal alterations in the expression of traditional vascular growth factors and their receptors (McClung et al., 2012) that coincide with the strain-dependent segregation of limb blood flow. Differentiating muscle cells secrete traditional vascular growth factors that act as both autocrine and paracrine factors to stimulate maturation in both endothelial and muscle cells (McClung et al., 2012, 2015; Mofarrahi et al., 2015) and represent a unique source of regenerative signals that could potentially be harnessed to improve the local ischemic microenvironment. Because a large proportion of murine pre-clinical limb ischemia work is performed in mice on either a mixed or largely BL6 background, regeneration from ischemic muscle myopathy is often masked or ignored.

**Figure 1:**
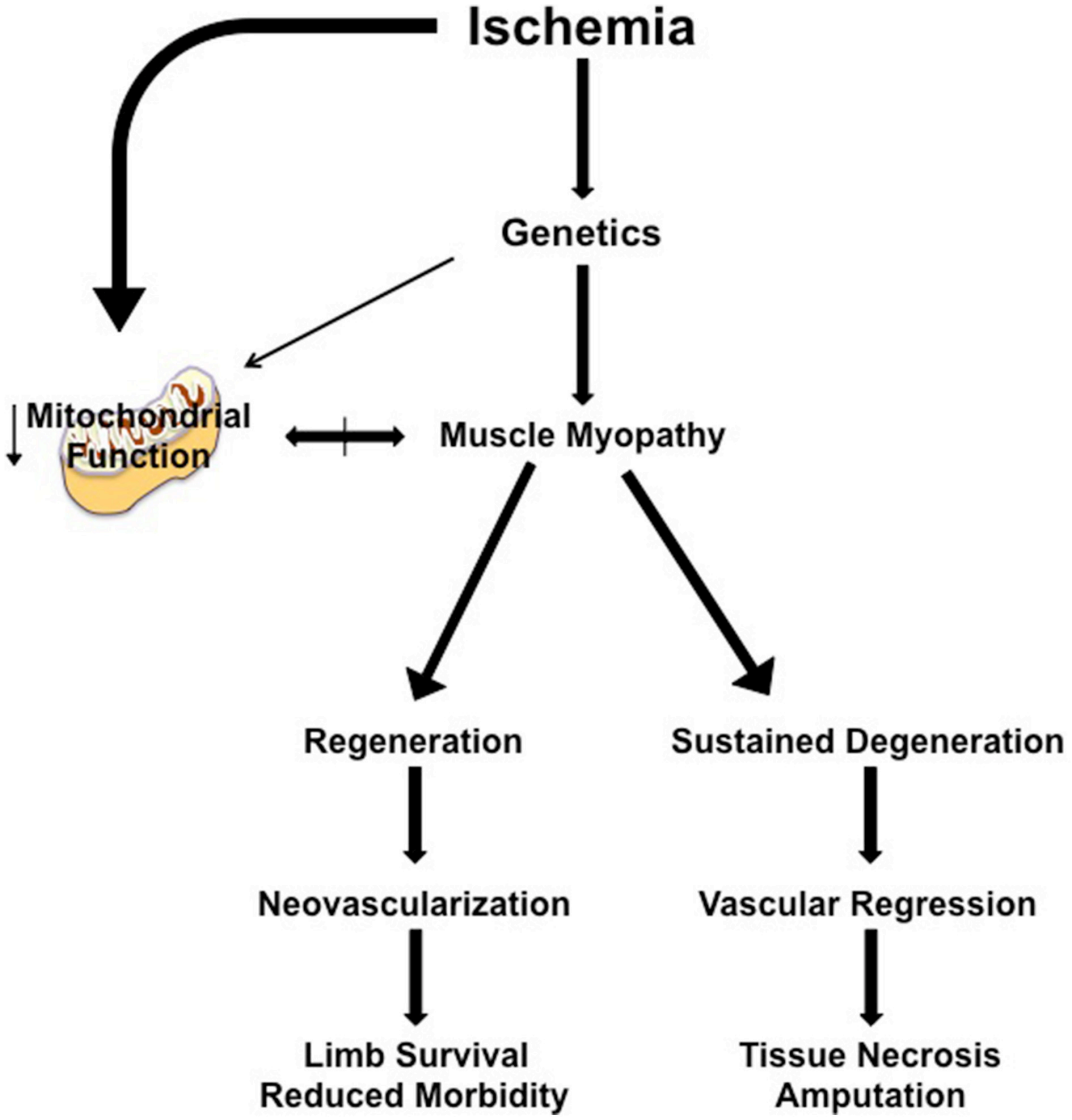
**Simplified model of the proposed role of muscle myopathy in the progression of limb pathology in critical limb ischemia**. Individual genetics play a role in determining the severity of the ischemic manifestation of limb pathology. Clinical interventions (endovascular or revascularization in nature) occur after the patient clinically presents with identifiable symptoms/manifestation of PAD, at a time when muscle myopathy is initiated or ongoing. Ischemic muscle myopathy involves muscle degeneration/regeneration cycles that: (1) function properly and result in a limb tissue microenvironment that is supportive of neovascularization and/or the clinical intervention, reducing morbidity and increasing the likelihood of limb survival, or (2) improperly function, resulting in a microenvironment that promotes continued degenerative myopathy and vascular regression that ultimately leads to tissue necrosis, morbidity, and secondary amputation. The role of mitochondrial function in ischemic limb muscle myopathy is not currently understood, but represents an exciting area for therapeutic exploration.

In a clinical CLI scenario, focusing on solely the vascular response is predicated on the idea that the ischemic muscle tissue is dispensable, at least short-term. Treatments that induce revascularization and/or nascent collateral vessel formation have proven ineffective to date (Annex, 2013; Hammer and Steiner, 2013; Cooke and Losordo, 2015) and indicate that a “restoration of flow approach” is not independently sufficient to rescue the limb. It is likely that myopathy and vasculopathy are interrelated components of a coordinated tissue response to CLI. Recent insights into the skeletal muscle response indicate that while the background genetics of an individual contributes to the density of pre-existing collateral vessels and the endogenous ability to generate nascent collateral vessels and capillaries, this simply isn't the sole determinant of pathology. The plasticity of the skeletal muscle facilitates temporal ischemic degeneration/regeneration in this environment, whereby genetically pre-determined deficits in muscle regenerative processes would result in cellular apoptosis and tissue necrosis that could negatively impact both endogenous neovascularization and/or the survival of a vessel graft. Simply put, limb muscle tissue that is already necrotic or beyond repair by endogenous regenerative mechanisms is representative of a local ischemic environment that is unable to sustain or promote neovascularization (Figure 1). Intricate coordination of therapies targeting muscle plasticity may be required to allow tissue survival and facilitate recovery until blood flow can be fully restored by surgical intervention and/or collateral vessel formation.

The importance of striated muscle to ischemic outcomes is readily accepted in cardiac ischemia/reperfusion, and there are numerous clinical trials involving therapeutic targeting of the cardiomyocyte (*clinicaltrials.gov*: NCT01502774, NCT01374321, NCT01172171, NCT00966563, NCT01572909). Therapies aimed at the skeletal muscle represent an untapped arena with great potential to advance the field of CLI research. Table 1 highlights the clinical work verifying limb skeletal muscle's role in PAD mortality over the last 10-years. Documented histochemical evidence of skeletal muscle myopathies and necrosis in PAD patients exists (Rissanen et al., 2002; Pipinos et al., 2007, 2008a); however the majority of the field operates under the assumption that myopathy is not important (Sealock et al., 2014). In stark contrast, muscle biologists studying neuromuscular diseases, dystrophy, or myofibrillar myopathies have embraced the contributions of the vasculature to the pathologic manifestations of their respective diseases. Abnormal skeletal muscle perfusion and resultant ischemia are believed to contribute to the pathology of Duchenne and Becker muscular dystrophies, and have spawned the “two hit (ischemia-metabolic stress) hypothesis” for muscle injury in these diseases (Asai et al., 2007). This hypothesis has driven approaches to treat these muscular dystrophies with phosphodiesterase-5 inhibitors like tadalafil, which improves blood flow, in an attempt to circumvent the ischemic component and improve muscle bioenergetics (Martin et al., 2012). Given the similarities between dystrophic myopathies and those identified with CLI, therapies aimed at skeletal muscle could be effective treatments for tissue degeneration and dysfunction during ischemia while also providing benefits to the vascular compartment of the affected limb. Interestingly, a common pathology linking neuromuscular disorders involving degeneration/regeneration is mitochondrial dysfunction (Katsetos et al., 2013). Mitochondria have recently garnered attention in the PAD literature (Brass, 2013; Hiatt et al., 2015) and may represent an evolutionarily conserved “starting point” for investigation into CLI myopathy.

**Table 1:** **Clinical studies implicating skeletal muscle function with mortality**.

**Study**	***n***	**Patient population**	**Skeletal muscle factors associated with mortality**
Gardner et al., 2008	434	PAD	6-min walk test, speed, and stair climbing scores
de Liefde et al., 2009	2191	PAD	Total treadmill walking distance
Singh et al., 2010	410	PAD	Attenuated knee extensor/flexion and hip extension strength in men, but not women
McDermott et al., 2011	440	PAD	Decline in 6-min walk test, and fast- and usual-paced 4-m walk test
McDermott et al., 2012	434	PAD	Lower calf muscle density and strength
Raval et al., 2012	425	PAD	Obesity associated with lower calf muscle density and greater declines in muscle density over time.
Jain et al., 2013	442	PAD	Walking speed and strain climbing scores from walking impairment questionnaire
Leeper et al., 2013	725	PAD	Symptom limited walking time on ramped treadmill test
Thompson et al., 2014	187	PAD	Calf muscle citrate synthase activity (marker of mitochondrial content)
Matsubara et al., 2015	64	CLI	5-year survival rate significantly lower in patients with sarcopenia (total body)

*A brief literature search using PUBMED, MEDLINE, and SCOPUS was conducted. Studies assessing the association between skeletal muscle health/function and cardiovascular/all-cause mortality are shown above with abbreviated summary of findings. PAD, peripheral arterial disease; CLI, critical limb ischemia; n, number of patients*.

## Skeletal muscle mitochondria in the ischemic limb

Mitochondria have numerous roles in the muscle cell, including the generation and maintenance of energy and redox charge, gatekeeping the mortality of ischemic cells (Karch and Molkentin, 2015; Shirihai et al., 2015), and the production of reactive oxygen species (ROS). Mitochondria also communicate with the rest of the cell through “signals” such as metabolites, cytochrome c release, and via redox-dependent cascades. Decreased muscle metabolism, impaired mitochondrial respiration, decreased expression of mitochondrial enzymes, increased oxidative stress, and somatic mutations in mitochondrial genes have been reported in limb muscle of patients' with PAD (Keller et al., 1985; Hands et al., 1986; Zatina et al., 1986; Bhat et al., 1999; Brass and Hiatt, 2000; Pipinos et al., 2000a, 2006, 2008b; Brass et al., 2004; Isbell et al., 2006; Schocke et al., 2008; Wurdeman et al., 2012; Weiss et al., 2013; Koutakis et al., 2014). Using non-invasive magnetic resonance spectroscopy, several labs have demonstrated that limb muscle from PAD patients' displays slower phosphocreatine (PCr) recovery, indicative of a lower muscle/mitochondrial oxidative capacity (Keller et al., 1985; Hands et al., 1986; Pipinos et al., 2000a,b; Isbell et al., 2006; Schocke et al., 2008). Interpretation of these data can be complicated by the influence of an intact but poorly functioning vascular system, perpetuating the idea that the reduced PCr recovery rates are more related to poor perfusion during the recovery period. Gastrocnemius muscle biopsies from PAD patients, however, also demonstrate reduced mitochondrial content and enzyme activity *ex vivo* (where oxygen delivery is not a limitation; Pipinos et al., 2003, 2006, 2007), and pre-clinical studies have recapitulated these findings (Pipinos et al., 2008b; Lejay et al., 2015). It is not currently known whether alterations in mitochondrial content or function cause ischemic muscle myopathy, but a recent report linked muscle mitochondrial content (reported as citrate synthase protein abundance) to PAD mortality (Thompson et al., 2014).

A lack of oxygen delivery to limb muscle tissue induces a progressive accumulation of ischemic injury that manifests as declining muscle function (Pipinos et al., 2007, 2008a; McDermott et al., 2012; Cluff et al., 2013; Weiss et al., 2013; Koutakis et al., 2014). A potential source for this tissue injury may be mitochondrial-derived ROS and the resulting oxidative stress with chronically elevated ROS. Pipinos et al. reported the first indirect evidence for skeletal muscle “oxidative stress” in patients with PAD (Pipinos et al., 2006). Recent work from this group suggests that these same indirect markers of oxidative stress may be related to disease severity (Fontaine Stage and ABI;Weiss et al., 2013). The potential also exists for repeated ischemia-reperfusion events in skeletal muscle from CLI patients (Lejay et al., 2014). When blood flow and pressure is low, arterial blockages may result in low oxygen tensions in muscle tissue that could be severe enough to inhibit mitochondrial complex IV (cytochrome c oxidase) and consequently electron flow in the electron transport system. This would result in the accumulation of metabolites and reducing equivalents (NADH and FADH_2_) that, upon re-oxygenation by surgical intervention or endogenous collateral flow with activity or mechanical loading, would be rapidly metabolized. These ischemia-reperfusion events have been well documented to produce large amounts of ROS in cardiac, brain, liver and renal tissues (Chouchani et al., 2014) and could be intermittently triggered by small amounts of physical activity or mechanical loading. For additional details on oxidative stress with PAD, we would recommend other excellent reviews (Brass, 1996; Pipinos et al., 2007, 2008a).

Because mitochondria are a major source of both reductive power (e.g., NADPH) and oxidants (superoxide anion and hydrogen peroxide), they serve as a metabolic rheostat controlling cellular redox homeostasis. Flux through both the reductive and oxidative arms contributes to redox signaling through redox modifications to cysteine residues that regulate the structure/function of target proteins (Go and Jones, 2013). Post-translational modifications such as S-nitrosylation, glutathionylation, sulfenylation, and disulfide bond formation are also considered mechanisms of redox signaling. Although the redox signaling field is at an early stage, recent studies suggest regulation of several cellular pathways relevant to the ischemic microenvironment including: muscle autophagy (Rahman et al., 2014), contractile dysfunction (reviewed in Powers et al., 2011), atrophy (Lawler et al., 2003), mitochondrial fission and fusion (reviewed in Willems et al., 2015), vascular growth and remodeling (reviewed in Bir et al., 2012), gene stability (Mikhed et al., 2015), and cellular proliferation and death (Wang et al., 2013; L'honoré et al., 2014). An oxidative shift with elevated ROS production in one cell type may have a direct and/or indirect effect on other resident cell types. Although it is difficult to imagine that charged, highly reactive oxygen/nitrogen species arising within subcellular organelles (e.g., mitochondria) or from cytosolic enzymes (e.g., xanthine oxidase) could escape the oxidant buffering systems (e.g., glutathione peroxidases, peroxiredoxins, superoxide dismutase, catalase) and travel to neighboring cells, ROS species, particularly those not carrying a charge (e.g., H_2_O_2_), produced by membrane bound enzymes (e.g., NADPH oxidase) may be capable of directly affecting nearby cells. It is likely that altered redox homeostasis in one cell would dramatically alter the local microenvironment through paracrine signaling. For example, skeletal muscle redox alterations have been shown to decrease endothelial cell angiogenic properties via the HIF-1α signaling cascade (Dromparis et al., 2014). Further, HIF-1α is a known transcriptional regulator for vascular endothelial growth factor (VEGF), which plays a vital role in angiogenesis (Rhoads et al., 2009).

## Endothelial mitochondria in the ischemic limb

Early research on the cellular bioenergetics of endothelial cells (ECs) suggested a heavy cellular reliance on glycolytic metabolism for the energy requirements of normal processes (Dobrina and Rossi, 1983; Leighton et al., 1987; Krützfeldt et al., 1990; Laing et al., 1992). These studies reported high activities of key enzymes in glycolytic metabolism (phosphofructokinase, hexokinase), high rates of lactate production in aerobic conditions, and low rates of glucose oxidation especially when high levels of glucose are present (Crabtree Effect). Additionally, ECs have a relatively low mitochondrial content, [less than 5% of the cell volume vs. 5–20% in skeletal muscle (Hoppeler et al., 1981; Groschner et al., 2012; Dahl et al., 2015; Jacobs et al., 2015)]. Some studies suggest mitochondrial ATP production is dispensable in ECs (Quintero et al., 2006) and there appears to be supportive evidence in that limb ECs are resistant to ischemic insult in CLI patients (Mertens et al., 1990; Noll et al., 1990). There is also a distinct body of research, however, indicating that mitochondria are critical organelles to the viability and function of ECs (Quintero et al., 2006; Goveia et al., 2014; Eelen et al., 2015). Mesenchymal stem cells form tunneling nanotubes that transfer mitochondria to ECs to rescue cellular aerobic respiration and stave off apoptosis induced by ischemia/reperfusion (Liu et al., 2014), a response that could be particularly important in stroke patients (Chan, 2005; Li et al., 2012; Lejay et al., 2014; Mishiro et al., 2014). Capillary EC mitochondrial cytopathies decrease angiogenesis and precede myofiber injury in early infants with mitochondrial diseases, a finding termed “mitochondrial angiopathy” (Sarnat et al., 2012). Overexpression of mitochondrial Thioredoxin-2 (Trx2) improves EC proliferation and arteriogenesis in the ischemic limb (Dai et al., 2009) and cancer researchers now utilize mitochondrial uncouplers in attempts to reduce tumor size due to their effects on neovascularization (Coutelle et al., 2014). These recent findings support an integral role for the mitochondria in the regulation of EC function and indicate this organelle's potential as a therapeutic target for CLI.

## Mitochondrial dynamics

Mitochondria are dynamic organelles that rely on complex signals orchestrating dynamic fission and fusion events believed to be responsible for regulating mitochondrial quality control. Fission and fusion are involved in the elimination of damaged/dysfunctional mitochondria (Song et al., 2015) which may serve as major sources of ROS. A cell's decision to remove dysfunctional mitochondria plays a vital role in limiting cellular damage/apoptosis while maintaining cell function. Damaged and depolarized mitochondria are targeted by PTEN-induced putative kinase 1 (PINK1), which drives Parkin-mediated mitophagic engulfment by autophagosomes (termed “mitophagy,” for detailed reviews see Dorn and Kitsis, 2015; Shirihai et al., 2015). Recent preclinical evidence suggests that defects in mitophagy exacerbate cardiomyocyte injury and decrease survival following ischemia/reperfusion (Song et al., 2014), indicating an increased cellular sensitivity to ischemic stress (Kubli et al., 2013). Mitophagy is critically important to the plasticity of skeletal muscle (Liesa and Shirihai, 2013) and is a unique process that could be similarly important to the health of the vasculature in the ischemic limb. Ischemia/reperfusion-induced impairments in EC mitochondrial respiratory capacity have been intricately linked to accelerated fission caused by excessive oxidative and nitrosative stress (Giedt et al., 2012). Moreover, siRNA-knockdown of mitofusin-1 or mitofusin-2 impairs EC angiogenic function *in vitro* and increases markers of apoptosis under stress (serum-deprivation; Lugus et al., 2011). There are no current investigations into the potential role of mitophagy in limb muscle pathology with CLI, although mitochondrial dynamics provide an attractive candidate for exploration. The accumulation of damaged mitochondria is likely to lead to increased ROS, an oxidative shift in the redox environment, and impaired energy production; all factors contributing to a pathologic ischemic microenvironment.

## Primary CLI risk factors associated with altered mitochondrial function

There are numerous risk factors linked to the CLI manifestation in PAD patients (Fowkes et al., 2013; Nehler et al., 2014). The two strongest risk factors for CLI, smoking and diabetes, are particularly provocative in terms of the subject matter of this review due to the ability of both to impair mitochondrial function in multiple cellular compartments of the ischemic limb.

### Smoking

From a physiologic perspective, smoking impairs microvascular reactivity (Ijzerman et al., 2003), increases intima-media carotid wall thickness (Howard et al., 1994), decreases flow-mediated dilation in the brachial artery (Langham et al., 2015) and increases the likelihood of atherosclerotic lesion formation (Yanbaeva et al., 2007). Pre-clinically, chronic cigarette smoke exposure severely alters vascular structure and function, including facilitating oxidative and nitrosative stress (Talukder et al., 2011). ECs exposed to cigarette smoke extract *in vitro* have lower mitochondrial integrity, rapid loss of mitochondrial membrane potential, and arrest of cell cycle progression (Henderson et al., 2008). Interference of the respiratory chain by either hydroquinone or carbon monoxide is believed to be a key component of smoking induced mitochondrial dysfunction in skeletal muscles (Degens et al., 2015), as well as impaired oxygen delivery due to carbon monoxide binding with hemoglobin/myoglobin. Interestingly, the combination of high-fat diet and nicotine results in increased oxidative stress and substantial lipid accumulation adjacent to swollen intramyofibrillar mitochondria in peripheral skeletal muscle (Sinha-Hikim et al., 2014). Taken together, the global cellular response to smoking demonstrates the potential for smoking to alter not only physiologic vessel function and the time-course of atherosclerotic lesion formation, but also the health of peripheral muscle cells. Interestingly, only a small series of research studies examine the muscle regenerative aspect, several of which are linked with healing rates after orthopedic surgery (Karim et al., 2006; Lundgreen et al., 2014; Mall et al., 2014). There are, however, a number of studies demonstrating ultrastructural and functional alterations in cardiomyocyte mitochondria after exposure to cigarette smoke (Yamada et al., 2009; Hu et al., 2013; Tippetts et al., 2014). Further work is necessitated to directly examine the effects of smoking on the mitochondria of the ischemic limb muscle, but this area represents an exciting arena with the potential to result in singular therapies for multiple co-morbidities associated with smoking and cardiovascular disease.

### Diabetes

Type II Diabetic patients with PAD are five times more likely to present clinically with CLI accompanied by tissue loss (Jude et al., 2001) and do not respond well to revascularization or endovascular interventions (Derubertis et al., 2008; Malmstedt et al., 2008). While diabetes may exacerbate the development of plaque blockages in the arteries, the impact of metabolic syndrome/diabetes on other tissue compartments has not been investigated in the context of CLI. As a common risk-factor, one explanation for the diabetic increase in PAD susceptibility could conceivably be exacerbated muscle damage originating from compromised mitochondrial function prior to the onset of ischemia. Diabetes both reduces skeletal muscle mitochondrial function (Kelley et al., 2002; Petersen et al., 2004; Bonnard et al., 2008) and increases mitochondrial fission, fragmentation, and ROS production in human venous ECs (Shenouda et al., 2011), indicating the potential for exacerbated ischemic responses in multiple cellular compartments of the ischemic limb. Chronic oxidative stress caused by nutrient oversupply to muscle mitochondria is implicated in reduced diabetic mitochondrial respiratory function (Bonnard et al., 2008; Anderson et al., 2009a,b), whereas mitochondrial-targeted antioxidants confer protection against diet-induced dysfunction (Hoehn et al., 2009; Anderson et al., 2009b; Lee et al., 2010). Taken together, these findings suggest the possibility that compromised muscle and endothelial mitochondrial function may be pre-conditioning the limb tissue to respond poorly to the ischemic insult in diabetic CLI patients, resulting in greater myopathy and sustained tissue degeneration regardless of genetic susceptibility.

## Conclusions and future directions

A critical barrier to developing therapeutic strategies to PAD has been a lack of understanding of the mechanisms underlying the etiology and pathology of PAD. While the cause of PAD is unquestionably occlusive arterial disease, the limited success of surgical and angiogenic treatments suggest that factors other than blood flow may significantly contribute to patient outcomes. Physiologically, angiogenesis and neovascularization are directed by the metabolic demand of the resident tissue. Simply put, the return of blood flow will have little effect if the limb tissue is beyond repair. In this review, we have highlighted recent trends in CLI research that suggest limb musculature may be a viable and potentially parallel therapeutic option for both the myopathy and vasculopathy of CLI. Furthermore, limb muscle and EC mitochondria provide attractive specific targets for novel therapeutic intervention.

## Sources of funding

JM supported by NIH/NHLBI R00HL103797 and R01HL125695, and a Brody Brothers Endowment Award, DB supported by NIH/NHLBI R01 HL123647 and R15 HL122922, PN supported by NIH/NIDDK R01 DK096907.

### Conflict of interest statement

David A. Brown has served as a consultant for Stealth BioTherapeutics, which is developing novel treatments for mitochondrial diseases. The other authors declare that the research was conducted in the absence of any commercial or financial relationships that could be construed as a potential conflict of interest.
